# Vertebral Artery Pseudoaneurysm in a Single‐Vessel Posterior Circulation With Chronic Contralateral Occlusion and Recent Methamphetamine Use

**DOI:** 10.1155/crra/5898856

**Published:** 2026-06-09

**Authors:** Daniel Reyes, Daniel Gridley

**Affiliations:** ^1^ University of Arizona College of Medicine-Phoenix, Phoenix, Arizona, USA, arizona.edu; ^2^ Department of Radiology, Valleywise Health Medical Center, Phoenix, Arizona, USA; ^3^ Department of Radiology, Creighton University School of Medicine, Phoenix, Arizona, USA, creighton.edu

**Keywords:** bilateral vertebral artery occlusion, CT angiography, dissecting aneurysm, pseudoaneurysm, substance abuse, vertebral artery dissection

## Abstract

Bilateral vertebral artery disease presents a critical risk for posterior circulation stroke due to impaired collateral flow. This report details an adult presenting with acute neck pain and visual disturbances. Computed tomography (CT) angiography revealed left vertebral artery dissection at the C2 level with a 0.5 cm dissecting pseudoaneurysm and complete occlusion of the right vertebral artery. Magnetic resonance imaging (MRI) demonstrated no acute infarction. Symptoms improved with conservative management, including antiplatelet therapy. The case highlights the importance of recognizing high‐risk bilateral vertebral artery pathology, understanding its imaging features, and considering substance abuse as a risk factor for arterial dissection. This case is notable for V3 dissecting pseudoaneurysm in the only functional vertebral artery in the setting of chronic contralateral occlusion and recent methamphetamine use, successfully managed conservatively.

## 1. Introduction

Vertebral artery dissection represents an uncommon but important cause of posterior circulation stroke in younger adults. Pseudoaneurysm formation is a complication of dissection that further increases the risk of thromboembolism and hemodynamic compromise [1]. Bilateral vertebral artery disease carries a particularly high risk [2] because occlusion or hypoplasia of one vertebral artery renders the contralateral vessel solely responsible for basilar circulation, and any superimposed pathology can potentially be catastrophic. Substance abuse, especially methamphetamine use, is an emerging contributor to cervical artery dissection through acute hypertension, vasospasm, and endothelial injury [3, 4]. We report the case of a middle‐aged patient with a left V3 segment vertebral artery dissection and pseudoaneurysm in what serves as the only functional vertebral artery due to chronic right vertebral artery occlusion and in the setting of recent methamphetamine use, highlighting computed tomography (CT) angiography findings and the rationale for conservative management in preserving posterior circulation perfusion.

## 2. Case Presentation

A patient in their 40s with hypertension, substance abuse, and prior cerebrovascular accident, with a 20‐pack‐year smoking history but no history of diabetes mellitus and no documented remote cervical trauma, presented from a drug rehabilitation facility with acute headache, posterior neck pain, and right eye visual changes. The patient last used methamphetamine and fentanyl 3 days before symptom onset. On neurologic examination, there was decreased right eye visual acuity, broad‐based gait ataxia, and a positive Romberg sign. The patient was hemodynamically stable with no evidence of acute neurologic deficit. Laboratory results were unremarkable aside from mildly elevated liver enzymes. Initial noncontrast head CT was negative for intracranial hemorrhage or territorial infarction. The patient reported a prior left vertebral artery aneurysm diagnosis as well as right vertebral artery occlusion diagnosed sometime in the preceding year, but it was lost to outpatient follow‐up due to factors related to the substance use disorder.

### 2.1. Imaging Findings

CT angiography of the head and neck was performed using a helical acquisition protocol from the vertex to the aortic arch following intravenous administration of 65 mL of iohexol (Omnipaque 350 mg/mL), with multiplanar maximum intensity projection (MIP) reconstructions in axial, coronal, and sagittal planes and three‐dimensional (3D) reformats generated on an independent workstation. MRI of the brain was performed without intravenous contrast using multiplanar multisequence imaging, including T2‐weighted and fluid‐attenuated inversion recovery (FLAIR) sequences, demonstrating no acute intracranial abnormality. CT angiography of the head and neck demonstrated focal dissection of the left vertebral artery at the C2 level, accompanied by a 0.5 cm pseudoaneurysm that appeared as an irregular, contrast‐filled outpouching on coronal and axial reformations (Figure 1). Three‐dimensional reconstruction further highlighted the pseudoaneurysm and showed complete right vertebral artery occlusion from its origin to the C1 vertebral body without opacification of distal segments (Figure 2). Flow through the basilar artery depended solely on the left vertebral artery. MRI brain revealed no acute infarction or intracranial hemorrhage. Anterior circulation vessels were patent and unremarkable.

**Figure 1:**
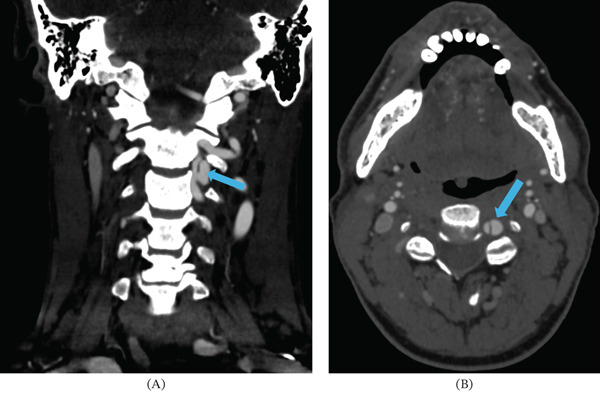
(A) Coronal and (B) axial computed tomography angiography (CTA) demonstrating dissection of the left vertebral artery at the level of C2 with associated pseudoaneurysm measuring 0.5 cm (blue arrows).

**Figure 2:**
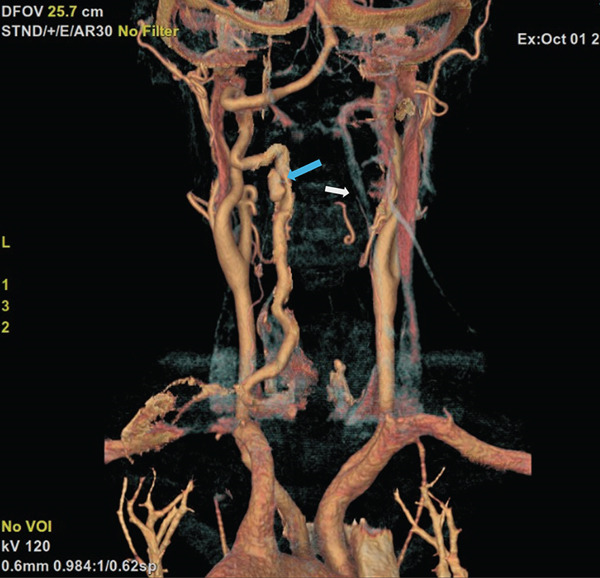
Computed tomography angiography (CTA) three‐dimensional (3D) reconstruction, right posterior lateral view of the neck, demonstrating dissection of the left vertebral artery with associated pseudoaneurysm measuring 0.5 cm (blue arrows). Occlusion of the right vertebral artery (white arrow) is shown from its origin to the level of the C1 vertebral body. Note: In this posterior lateral 3D projection, the patient′s left side is displayed on the left of the image, and the patient′s right side is displayed on the right of the image; this nonstandard orientation was selected to optimally visualize the pseudoaneurysm.

### 2.2. Diagnosis

The diagnosis for this patient included left vertebral artery dissection with a 0.5 cm pseudoaneurysm at the level of C2, chronic right vertebral artery occlusion, and bilateral vertebral artery disease.

Differential diagnostic considerations include atherosclerotic stenosis, fibromuscular dysplasia, vasculitis, and traumatic dissection. Atherosclerotic stenosis typically demonstrates concentric calcified plaque and smooth luminal narrowing at vessel origins without intramural hematoma or intimal flap. Fibromuscular dysplasia is characterized by the classic “string of beads” appearance without a surrounding hematoma. Vasculitis may cause smooth tapered multifocal narrowing, typically bilateral and lacking the eccentric pseudoaneurysm morphology seen here. Traumatic dissection shares imaging features with spontaneous dissection but is distinguished by clinical history. In this case, the narrow‐necked, irregular pseudoaneurysm, continuous with the parent lumen, eccentric contour, and absence of calcification, and clinical context of methamphetamine use strongly favored spontaneous dissection.

## 3. Discussion

Vertebral artery dissection presents when there is an intimal or medial tear, permitting blood to enter the vessel wall and form an intramural hematoma, which may result in luminal narrowing, occlusion, or pseudoaneurysm formation [5]. The incidence of cervical artery dissection in the general population is low, estimated at approximately 2.6 per 100,000 population per year, and has risen due to widespread use of advanced CT and magnetic resonance (MR) angiography imaging techniques that allow for improved diagnosis [5–7]. The V3 (C2 to dural entry) segment is the most frequently affected due to mechanical stress during head rotation and extension [8].

Vertebral artery pseudoaneurysms represent partial‐thickness vessel wall disruption with contained hemorrhage, lacking a true arterial wall and instead bounded only by media or adventitia and surrounding tissue [1]. On imaging, unlike true aneurysms, pseudoaneurysms are characterized by a narrow neck, irregular contours, surrounding hematoma, and contrast filling that is continuous with the parent vessel [1, 9]. Multidetector CT angiography (MDCTA) demonstrates luminal narrowing, intimal flap formation, pseudoaneurysm outpouchings, and vessel wall hematoma. MRI with fat‐saturated T1‐weighted sequences can reveal crescentic T1 hyperintensity representing subacute intramural hematoma, while conventional Doppler ultrasound has limited sensitivity for detecting dissections, particularly those causing low‐grade stenosis [1, 9, 10]. CT angiography offers high spatial resolution and rapid, noninvasive detection of vertebral artery pathology and is preferred for initial evaluation [1, 9]. The presence of key findings such as luminal narrowing, intimal flap, double lumen, vessel wall thickening, and pseudoaneurysm formation should prompt a search for additional lesions, especially bilateral disease [1, 9].

Clinical consequences include thromboembolism, which can result from mural thrombus and represents the most common cause of stroke in vertebral dissection and, less commonly, rupture [1, 5, 8]. Bilateral vertebral artery disease is a critical risk factor for posterior circulation stroke, as collateral flow is compromised; hypoplasia or occlusion of a vertebral artery increases dependency on the contralateral vessel and raises the risk of infarction markedly if the remaining artery is also diseased [2].

Substance abuse, especially methamphetamine, is an emerging risk factor for arterial dissection. Amphetamines increase vessel wall stress through acute hypertension, vasospasm, and direct endothelial toxicity [3, 4]. Several reports link amphetamine use temporally to vertebral artery dissection; this patient′s history of methamphetamine abuse may be contributory, suggesting the need for screening and education in at‐risk populations [2]. Notably, the patient had no documented history of diabetes mellitus and denied a history of remote cervical trauma. While vasculitis was considered in the differential diagnosis, no formal vasculitis workup was recorded on prior visits, and the acuity of presentation in the setting of recent methamphetamine use, combined with the absence of systemic inflammatory features, favored a substance‐related mechanism over vasculitic etiology. No formal vasculitis, connective tissue disorder, or hypercoagulable state workup was performed during this hospitalization, with laboratory investigations limited to complete blood count, comprehensive metabolic panel, prothrombin time/INR, and serologic screens for syphilis, hepatitis, and sexually transmitted infections. While the absence of such a workup represents a limitation, it is unclear whether the patient underwent such an investigation, either outpatient or at another facility, and in either case, the overall clinical picture, including the acute presentation immediately following recent methamphetamine use, absence of systemic inflammatory signs, and prior cardiac history consistent with long‐standing substance‐related vasculopathy, supported a methamphetamine‐related mechanism as the most plausible etiology.

Comparison with similar published cases contextualizes the management decision in this report. Winsløw et al. [3] described vertebral artery dissection at the V3 segment in a 37‐year‐old male with amphetamine abuse presenting with neck pain and vertigo, whose dissection resolved with dual antiplatelet therapy, a presentation closely mirroring our case, though that patient had a patent contralateral vertebral artery, unlike the single‐vessel posterior circulation in our patient. McIntosh et al. [4] reported methamphetamine‐associated carotid artery dissection with stroke, reinforcing the cerebrovascular toxicity of amphetamines across multiple vascular territories. Published cases of pseudoaneurysm involving a dominant or sole vertebral artery have most commonly been managed endovascularly with covered stent placement or coiling [1]. Our case contributes to the limited literature suggesting that conservative management with antiplatelet therapy may be appropriate in carefully selected patients, specifically those with small extracranial pseudoaneurysms, no infarction, neurological improvement, and hemodynamic stability, even in the high‐risk setting of a single‐vessel posterior circulation.

Management is still debated, with recent meta‐analyses finding no major difference in stroke prevention between antiplatelet therapy and anticoagulation for cervical artery dissection. However, antiplatelets may be favored when pseudoaneurysms are present to protect against aneurysm expansion [1, 8, 10]. Endovascular or surgical intervention is reserved for cases with recurrent symptoms, enlarging pseudoaneurysms, or high rupture risk, especially if symptomatic hypoperfusion is present [1, 8, 10]. Our patient improved with conservative treatment and antiplatelet therapy; the small, stable extracranial V3 pseudoaneurysm with preserved basilar perfusion and in the absence of infarction in a single‐vessel posterior circulation supported conservative management with antiplatelet therapy rather than immediate endovascular intervention. The CADISS trial, the largest randomized controlled trial comparing antiplatelet therapy to anticoagulation in cervical artery dissection, found no significant difference in stroke or death at 3 months, supporting the use of antiplatelets as a safe first‐line option in extracranial dissection [8]. In this patient, opting for conservative management was supported by the pseudoaneurysm being small (0.5 cm), extracranial, and in the V3 segment, no acute infarction identified on MRI, mild neurologic symptoms that improved with initial conservative care, and the patient remaining hemodynamically stable throughout the hospital course. Neurology recommended daily 81 mg aspirin without further endovascular workup. Although the single‐vessel posterior circulation raised concern for potential catastrophic consequences, including thromboembolism or pseudoaneurysm expansion, a watchful approach was taken in light of the patient′s hemodynamic stability and lack of active ischemia, with close imaging follow‐up outpatient planned following discharge.

Careful follow‐up imaging is important to assess stability, pseudoaneurysm size, and collateral circulation. Long‐term recurrence risk with new dissection is approximately 2% in the first month, declining to 1% annually thereafter [10]. Emphasis should be placed on aggressive cardiovascular risk factor reduction and substance abuse cessation [4].

This case is distinctive because the dissecting pseudoaneurysm arose in the only functional vertebral artery, with the contralateral vessel chronically occluded, creating a single‐vessel posterior circulation at risk for both thromboembolism and hemodynamic compromise. Additionally, the patient′s recent methamphetamine use is a plausible precipitating factor through acute hypertension, vasospasm, and endothelial injury, consistent with emerging reports linking amphetamines to cervical artery dissection [2–4].

## 4. Conclusion

This case highlights the importance of recognizing bilateral vertebral artery disease and single‐vessel posterior circulation, particularly when associated with substance abuse, as a prominent stroke risk factor in younger adults. CT angiography is essential for diagnosis and delineating pseudoaneurysm morphology, assessing collateral flow, and guiding management, particularly the choice between conservative and endovascular management. While extracranial vertebral pseudoaneurysms typically have a benign prognosis, bilateral vertebral pathology demands close follow‐up and risk factor reduction. In patients with a dissecting pseudoaneurysm in the only functional vertebral artery, careful imaging follow‐up and aggressive cardiovascular and substance use risk factor modification may allow safe conservative management. Clinicians should maintain heightened vigilance for stroke symptoms in similar patients and coordinate interdisciplinary management.

## Funding

No funding was received for this manuscript.

## Disclosure

None of the authors received outside funding for the production of this original manuscript, and no part of this article has been previously published elsewhere.

## Ethics Statement

The study was conducted in accordance with the Declaration of Helsinki. Ethical review and approval of this report were not required due to the retrospective nature of the report. This work is not considered human subjects research. The patient′s information has been deidentified, and informed consent has been obtained from the patient for this publication. This case report was prepared in accordance with the CARE (Case Report) guidelines.

## Conflicts of Interest

The authors declare no conflicts of interest.

## Data Availability

Data sharing is not applicable to this article as no datasets were generated or analyzed during the current study.

## Appendix A Questions

1. What is the most common mechanism of stroke in vertebral artery dissection?

A. Subarachnoid hemorrhage from pseudoaneurysm rupture
B. Hemodynamic hypoperfusion from stenosis
C. Thromboembolism from mural thrombus
D. Direct vessel occlusion at the dissection site

Answer: C

2. What is the most specific CT angiography finding for arterial dissection as opposed to atherosclerotic disease?

A. Pseudoaneurysm with a narrow neck and irregular contour
B. Concentric calcified plaque
C. Bilateral symmetric narrowing
D. Smooth luminal narrowing

Answer: A

3. Which risk factor in this case is emerging as potentially important in young adult vertebral artery dissection?

A. Diabetes mellitus
B. Amphetamine use
C. Hyperlipidemia
D. Connective tissue disease

Answer: B

4. T/F: Endovascular treatment is immediately indicated for all vertebral pseudoaneurysms.

Answer: F

5. T/F: Color Doppler ultrasound is the most sensitive method for detecting vertebral pseudoaneurysm.

Answer: F
